# Perception of emotional valence in horse whinnies

**DOI:** 10.1186/s12983-017-0193-1

**Published:** 2017-02-11

**Authors:** Elodie F. Briefer, Roi Mandel, Anne-Laure Maigrot, Sabrina Briefer Freymond, Iris Bachmann, Edna Hillmann

**Affiliations:** 1 Institute of Agricultural Sciences, ETH Zürich, Universitätstrasse 2, 8092 Zürich, Switzerland; 2 Koret School of Veterinary Medicine, Robert H. Smith Faculty of Agriculture, Food and Environment, the Hebrew University, Rehovot, 76100 Israel; 3 Division of Animal Welfare, Veterinary Public Health Institute, Vetsuisse Faculty, University of Bern, Länggassstrasse 120, 3012 Bern, Switzerland; 4 Agroscope, Swiss National Stud Farm, Les Longs Prés, 1580 Avenches, Switzerland

**Keywords:** Emotional contagion, *Equus caballus*, Emotion expression, Familiarity, Playbacks, Vocalisations

## Abstract

**Background:**

Non-human animals often produce different types of vocalisations in negative and positive contexts (i.e. different valence), similar to humans, in which crying is associated with negative emotions and laughter is associated with positive ones. However, some types of vocalisations (e.g. contact calls, human speech) can be produced in both negative and positive contexts, and changes in valence are only accompanied by slight structural differences. Although such acoustically graded signals associated with opposite valence have been highlighted in some species, it is not known if conspecifics discriminate them, and if contagion of emotional valence occurs as a result. We tested whether domestic horses perceive, and are affected by, the emotional valence of whinnies produced by both familiar and unfamiliar conspecifics. We measured physiological and behavioural reactions to whinnies recorded during emotionally negative (social separation) and positive (social reunion) situations.

**Results:**

We show that horses perceive acoustic cues to both valence and familiarity present in whinnies. They reacted differently (respiration rate, head movements, height of the head and latency to respond) to separation and reunion whinnies when produced by familiar, but not unfamiliar individuals. They were also more emotionally aroused (shorter inter-pulse intervals and higher locomotion) when hearing unfamiliar compared to familiar whinnies. In addition, the acoustic parameters of separation and reunion whinnies affected the physiology and behaviour of conspecifics in a continuous way. However, we did not find clear evidence for contagion of emotional valence.

**Conclusions:**

Horses are thus able to perceive changes linked to emotional valence within a given vocalisation type, similar to perception of affective prosody in humans. Whinnies produced in either separation or reunion situations seem to constitute acoustically graded variants with distinct functions, enabling horses to increase their apparent vocal repertoire size.

**Electronic supplementary material:**

The online version of this article (doi:10.1186/s12983-017-0193-1) contains supplementary material, which is available to authorized users.

## Background

Emotions are intense, short-lived affective reactions to specific events or stimuli. They can be characterised using two main dimensions (dimensional approach): valence (negative/unpleasant or positive/pleasant) and arousal (bodily activation or excitation; e.g. calm versus excited) [1]. Emotional arousal can be considered as the intensity of bipolar valence, which comprises the defensive (negative valence) and the appetitive (positive valence) motivational systems described in humans and other species [2].

Emotions can be transmitted through olfactory signals (pheromones present in conspecifics’ urine [3, 4]), visual signals (facial expressions [5]), acoustic signals [6, 7] or a combination of these [8, 9]. Perception of emotion expression can potentially induce the same emotion in the receiver as in the producer of the signal. This phenomenon is termed “state matching” or “emotional contagion”, and is the basis of empathy [10, 11]. For example, a signal indicating a high-arousal state could increase the emotional arousal of receivers (i.e. contagion of emotional arousal). If this signal is positive, it could also trigger a change in emotional valence from negative or neutral to positive (and vice-versa for negative signals) in receivers (i.e. contagion of emotional valence). Unlike higher, cognitive forms of empathy (e.g. sympathetic concern), the transmission of emotions from one individual to another is widespread in the animal kingdom [12]. It is enhanced by social closeness, familiarity and similarity between partners [10, 13], improves information transfer through state sharing between individuals, and results in higher coordination among group members and stronger inter-individual bonds [10, 14]. Because vocalisations are a very effective communication system (e.g. they can be transmitted over long distances, around obstacles, and can be perceived in low visibility conditions [15]), they constitute a rapid means of transmitting information to conspecifics and are, as a result, a prominent channel for emotional contagion [16].

Variation in the structure of vocalisations associated with emotional valence and arousal (i.e. vocal expression of emotions) have been observed across species [17]. While both changes in call types (i.e. discrete calls, e.g. pig, *Sus scrofa*, grunts to squeals [18]) and modification in the acoustic structure of a given call type (i.e. graded calls, e.g. meerkat, *Suricata suricatta*, alarm calls [19]) have been observed with variation in the emotional arousal experienced by the producer, contexts of opposite valence are usually associated with different call types ([17, 20] e.g. change from horse, *Equus caballus*, whinnies to squeals, from dog, *Canis lupus familiaris*, bark to growl, or from human laughter to crying). However, acoustic variation within call types that are produced in both negative and positive situations (e.g. contact calls) can also occur (e.g. African elephant, *Loxodonta Africana*, rumbles [21]; bonobos, *Pan paniscus*, peeps [22]; goat, *Capra hircus*, bleats [23]; horse whinnies [24]).

Perception of the variation existing within specific call types as a function of the emotional arousal of the producer has been mainly studied in non-human animals in alarm contexts. These studies revealed that conspecifics respond more to alarm calls that have been artificially modified to mimic higher urgency levels (i.e. the parameters indicating urgency have been increased [25–28]). The ability to perceive indicators of arousal in other types of calls has also been shown (e.g. [29–31]). Additionally, clear evidence for vocal contagion of emotional arousal (i.e. matching between the emotion of the producer and the receiver) exists in zebra finches (*Taeniopygia guttata*); females show raised corticosterone levels when hearing distance calls emitted by their pair mate given orally administered exogenous corticosterone, compared to when hearing regular distance calls [32]. However, to our knowledge, it is not known if receivers are able to perceive variation occurring within a given type of vocalisation as a function of the emotional valence of the producer, and if emotional contagion occurs as a result. This ability could allow species with limited vocal repertoires to communicate different emotions using the same vocalisation type. Such acoustically graded variants could, as a result, be associated with different functions (e.g. trigger retreat or approach), in the same way as different call types, and might be as important as call-type differentiation for modulating social interactions [7, 33, 34].

We investigated if domestic horses can perceive indicators of emotional valence in whinnies of familiar and non-familiar conspecifics, independently of the context of reception (i.e. using only the acoustic features of whinnies), and if contagion of emotional valence occurs. As a highly social species [35], horses should benefit from acoustic perception of emotions, in order to regulate social interactions within harems (stallion, females and foals) or bachelor bands (young or old stallions without a harem) [35]. Eight call types have been described in this species: whinnies, nickers, squeals, blows, snores, snorts, roars, and groans [36, 37]. Whinnies provide information about sex, body size and individuality [38], reproductive success [39] and emotions (valence and arousal [24]), while squeals provide information about dominance status [40]. Conspecific receivers can decipher familiarity [38, 41] and stallion fertility [39] encoded in whinnies, as well as dominance status encoded in squeals [40]. Furthermore, horses are capable of cross-modal individual recognition of conspecifics, matching whinnies to visual/olfactory characteristics of the caller [42].

Whinnies are the most common call type produced by horses and can be emitted in both negative and positive contexts (e.g. separation and reunion with conspecifics, anticipation of both unpleasant and pleasant events, disturbances, frustration and curiosity [36]). Our previous study revealed that these calls are constituted by two fundamental frequencies (“F0” and “G0”, suggesting biphonation), and that whinnies produced during social separation from either one or all group members (negative situations) are longer and have a higher G0 frequency than those produced during social reunion with one or all group members (positive situations) [24]. Separation and reunion whinnies thus constitute acoustically graded variants of the same call type. The negative and positive situations were also characterised by different behavioural responses in the producer; horses displayed less chewing motion (moving the lower jaw up and down without food [43]), and spent more time with the head high in the negative compared to the positive situation [24]. Here, we tested if information about emotional valence in whinnies can be deciphered by both familiar and unfamiliar conspecifics using playback experiments. We predicted that horses would show different physiological and behavioural responses to negative and positive whinnies, therefore validating emotion perception. If contagion of emotional valence occurs, we expected horses to display more behavioural indicators of negative emotions (head high) during playbacks of negative whinnies, and more behavioural indicators of positive emotions (chewing motion) during playbacks of positive whinnies (i.e. state matching between producer and receiver [12]). We also expected the acoustic features of whinnies to affect the responses of receivers in a graded way, with the time spent chewing decreasing and the time spent with the head high increasing with an increase in the duration and G0 of the calls played back, as predicted with a change from negative to positive emotions. As the acoustic channel is the main channel of communication in humans (speech), the study of vocal contagion of emotions in non-human animals is a promising way to understand the evolution of emotional contagion and empathy [10].

## Results

We tested 18 horses of various breeds (Additional file 1) housed in five different farms with four playback treatments each: 1) separation (negative) whinnies from a familiar horse, 2) reunion (positive) whinnies from the same familiar horse, 3) separation (negative) whinnies from an unfamiliar horse, and 4) unfamiliar reunion (positive) whinnies from the same unfamiliar horse. Each playback consisted in three whinnies produced by the same horse. Subjects were tested with the four treatments over two consecutive days (two playbacks per day). The order of the treatments was counterbalanced within horses for valence and between horses for familiarity. Familiar whinnies were recorded from horses housed in the same farm as the subjects, while unfamiliar whinnies were recorded from horses housed in other farms. Separation whinnies were produced by horses during separation from either one or all the other horses from their farm (“group members”). Reunion whinnies were produced when these horses were reunited with one or all group members, following the separation situation. Horses are highly gregarious animals and separation from conspecifics is thus stressful for them (i.e. emotionally negative), while their motivation to reunite with conspecifics is high (i.e. emotionally positive) [35, 44]. Separation whinnies were thus assumed to be of negative valence, and reunion whinnies of positive valence [24]. In order to investigate if horses could perceive vocal indicators of valence independently of the context of reception (i.e. if valence cues are stimulus-independent), horses were tested in their home environment (“neutral” context). We measured both their physiological and behavioural responses to each whinny played back. We analysed three physiological and five behavioural parameters that were previously shown to be affected by emotional valence and/or arousal [24], in addition to the latency of the subjects to respond to the playbacks (Table 1). We then included all these parameters in a principal component analysis to eliminate redundancy. We tested the effect of the valence and familiarity of the whinnies played back, and of the interaction between these two factors, on the scores of the resulting principal components (PC) with eigenvalue greater than 1 using linear-mixed effects models (LMMs). As responses to the playbacks are likely to be affected by the sex of the producer in respect to the sex of the subject, we also included a factor indicating whether the whinnies played back were produced by a horse of the same sex as the subject or not. Interactions between this factor and valence and familiarity were also fitted in the models.

**Table 1:** Abbreviations and descriptions of the physiological, behavioural and vocal parameters measured

	Abbreviation	Description	Arousal/Valence
Physiology	RR (ms)	Inter-heart-beat interval	**A**
	RespRate (breaths/s)	Respiration rate	**A**
	SkinT (°C)	Skin temperature	V + A
Behaviour	Locomotion	Proportion of time spent moving (walk, trot or canter)	**A**
	HeadMov (min-1)	Number of rapid head movements per minute	V + A
	HeadHigh	Proportion of time spent with the eye line above the tip of the shoulder	**V**
	Chewing	Proportion of time spent chewing (i.e. moving the lower jaw up and down in a chewing motion). This behaviour is performed without the presence of food in the mouth	**V** + A
	VocRate (min-1)	Number of vocalisations (whinnies or nickers) per minute	V (nickers)
	LatenceRes	Latency from the onset of the call played back to the first behavioural response (including all the above described behaviours)	-
Vocalisations	Dur (s)	Duration of the whinny	**V** + A
	G0Start (Hz)	Frequency value of G0 at the start of the whinny	**V** + A
	G0Max (Hz)	Maximum G0 frequency value across the whinny	V + A
	G0Mean (Hz)	Mean G0 frequency value across the whinny	**V** + A
	F0Start (Hz)	Frequency value of F0 at the start of the whinny	**A**
	F0Max (Hz)	Maximum F0 frequency value across the whinny	V + A
	F0Mean (Hz)	Mean F0 frequency value across the whinny	A
	AMVar (dB/s)	Cumulative variation in amplitude divided by the total whinny duration	V + A
	AMExtent (dB)	Mean peak-to-peak variation of each amplitude modulation	V + A
	Q25% (Hz)	Frequency value at the upper limit of the first quartiles of energy	V + A
	Q50% (Hz)	Frequency value at the upper limit of the second quartiles of energy	V + **A**
	Q75% (Hz)	Frequency value at the upper limit of the third quartiles of energy	V + **A**

Whether each parameter was significantly affected by emotional valence (V) or arousal (A) in our previous study [24] is indicated. Bold “V” indicates reliable cues to valence, i.e. parameters that were changing consistently with valence and were clearly more affected by valence than arousal. Bold “A” indicates reliable cues to arousal, i.e. parameters that were changing consistently with arousal and were clearly more affected by arousal than valence [24]

**Fig. 1:**
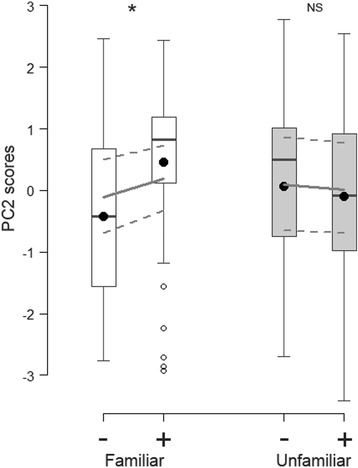
Response of the horses to the playbacks. Scores of the second principal component (PC2) of the principal component analysis as a function of the four playback treatments (familiar (*white*)/unfamiliar (*grey*) * separation (-)/reunion (+); box plot: the horizontal line shows the median, the box extends from the lower to the upper quartile and the whiskers to 1.5 times the interquartile range above the upper quartile or below the lower quartile; open circles indicate outliers and black circles the mean; the grey lines show the model estimates (continuous line) and 95% confidence intervals (*dashed lines*)). More positive PC2 scores corresponded to horses that moved their head more, had their head high for a longer duration, responded faster and had a slower respiration rate (Table 2) (Tukey post-hoc test: * *p* < 0.05, NS = Non-Significant)

Familiarity influenced PC1 scores (PC1: 27.24% of the variance, Table 2; LMM: *N* = 18 horses, *P* = 0.022; *R* ^*2*^_GLMM(m)_ = 2.94%, *R* ^*2*^_GLMM(c)_ = 59.68%); horses had shorter inter-pulse-intervals (RR; i.e. faster heart rates), moved more (Locomotion), moved their head more (HeadMov), had their head high for a longer duration (HeadHigh), vocalised more (VocRate) and responded faster (LatenceRes; i.e. any change in behaviour after the onset of the playback) when hearing unfamiliar whinnies (model estimates for PC1 score: mean [95% confidence interval] = -0.04 [-0.59, 0.55]) compared to familiar whinnies (-0.31 [-0.91, 0.35]). In addition, the interaction between valence and familiarity influenced PC2 scores (PC2: 20.56% of the variance, Table 2; LMM: *N* = 18 horses, *P* = 0.044; *R* ^*2*^_GLMM(m)_ = 5.90%, *R* ^*2*^_GLMM(c)_ = 39.90%). Post-hoc pairwise comparisons showed that PC2 scores differed between separation and reunion whinnies when these were familiar to the subject (Tukey post-hoc test: *Z* = 2.87, *P* = 0.021, *N* = 18 horses; *R* ^*2*^_GLMM(m)_ = 11.86%, *R* ^*2*^_GLMM(c)_ =46.95%), but not when they were unfamiliar (Tukey post-hoc test: *Z* = -0.74, *P* = 0.88, *N* = 18 horses, *R* ^*2*^_GLMM(m)_ = 0.70%, *R* ^*2*^_GLMM(c)_ =16.23%); horses had lower respiration rates (RespRate), moved their head more (HeadMov), had their head high for a longer duration (HeadHigh) and responded faster (LatenceRes) when hearing familiar reunion compared to familiar separation whinnies (Table 2; Fig. 1). The effects of valence, familiarity, sex of the horses or interactions between these factors on PC1 to PC3 not mentioned above were not significant (see Additional file 2 for statistical results of these factors and Additional file 3 for model estimates).

**Table 2:** Loadings of the physiological and behavioural parameters measured during the playbacks on the principal components with eigenvalue > 1 (PC1 to PC3 on a total of 9)

		Principal components		
	Parameters	PC1	PC2	PC3
Physiology	RR	**-0.74**	0.36	-0.08
	RespRate	0.37	**-0.58**	-0.15
	SkinT	0.38	-0.09	-0.39
Behaviour	Locomotion	**0.74**	-0.35	0.05
	HeadMov	**0.48**	**0.51**	0.20
	HeadHigh	**0.40**	**0.72**	0.02
	Chewing	0.03	0.15	**-0.89**
	VocRate	**0.60**	-0.23	0.11
	LatenceRes	**-0.58**	**-0.64**	0.05
Eigenvalue		**1.57**	**1.36**	**1.02**
% variance		**27.24**	**20.56**	**11.49**

Bold types indicate the heaviest factor loadings (|*r*| > 0.40). Eigenvalues and variances explained are given at the bottom of the table (see Table 1 for abbreviation of the parameters)

In order to test if separation and reunion whinnies also affected the responses of the horses in a continuous way, we tested the effect of the vocal parameters of the whinnies played back on the responses of the horses. To this aim, the calls played back were analysed by measuring vocal parameters previously shown to be affected by emotional valence and/or arousal ([24] Table 1). These parameters were included in a second principal component analysis to eliminate redundancy. The effect of the scores of the extracted principal components (PCv; eigenvalue > 1) on the PC scores corresponding to the physiological and behavioural responses of the subjects to the calls played back was then tested using LMMs. PC3v, which explained 15.27% of the variance in the vocal parameters of the calls played back (Table 3), influenced PC2 scores (LMM: *N* = 18 horses, *P* = 0.022; *R* ^*2*^_GLMM(m)_ = 3.54%, *R* ^*2*^_GLMM(c)_ = 39.33%); horses had slower respiration rates (RespRate), moved their head more (MovHead), had their head high for a longer duration (HeadHigh) and responded faster (LatencyRes; PC2, Table 2) when whinnies played back to them had lower fundamental frequencies (G0: G0Start, G0Max and G0Mean; and F0: F0Start and F0Max), were less modulated in amplitude (AMVar) and had a higher first quartile of energy (Q25%) (PC3v; Table 3; slope estimate ± SE: -0.16 ± 0.06). The effects of PC1v to PC4v on PC1 to PC3 not mentioned above were not significant (see Additional file 4 for statistical results of these factors, including slope estimates ± SE).

**Table 3:** Loadings of the vocal parameters extracted from the calls played back on the principal components with eigenvalue > 1 (PC1v to PC4v on a total of 12)

	Principal components			
Parameters	PC1v	PC2v	PC3v	PC4v
Dur	0.19	**0.64**	0.28	-0.14
G0Start	**-0.61**	0.20	**0.61**	-0.19
G0Max	**-0.81**	-0.11	**0.48**	0.02
G0Mean	**-0.81**	-0.13	**0.51**	0.01
F0Start	**0.56**	**0.62**	**0.41**	0.11
F0Max	**0.61**	**0.60**	**0.44**	0.04
F0Mean	0.30	**0.81**	-0.08	0.06
AMVar	**0.45**	-0.34	**0.44**	**0.54**
AMExtent	0.13	-0.36	0.15	**0.83**
Q25%	**-0.41**	**0.58**	**-0.50**	**0.41**
Q50%	**-0.73**	**0.52**	-0.20	0.31
Q75%	**-0.76**	**0.42**	-0.09	0.17
Eigenvalue	**1.99**	**1.70**	**1.35**	**1.17**
Cum % variance	**33.16**	**24.11**	**15.27**	**11.34**

Bold types indicate the heaviest factor loadings (|r| > 0.40). Eigenvalues and variances explained are given at the bottom of the table (see Table 1 for abbreviation of the parameters)

## Discussion

Using playback experiments, we tested if horses are able to discriminate whinnies produced during separation and reunion by both familiar and unfamiliar conspecifics, independently of the context (i.e. using only the acoustic features of the calls), as well as whether contagion of emotional valence occurs. Our results showed that horses reacted differently to separation and reunion whinnies when these calls were produced by familiar horses, but not when they were produced by unfamiliar individuals. In addition, some parameters of the whinnies played back, which had been previously shown to differ between separation and reunion situations ([24] F0, G0 and Q25%), affected the responses of the horses in a continuous way. This suggests that these two types of whinnies are graded into one another not only in their production [24], but also in the way they affect conspecifics. As the number of reliable indicators of emotions (revealed during our previous study) was limited (two indicators: chewing motion, time spent with head high [24]), we did not find evidence for negative emotions during playbacks of separation whinnies nor for positive emotions during playbacks of reunion whinnies. It is thus unclear whether contagion of emotional valence occurred. However, our study shows that horses are capable of perceiving variation in vocal parameters indicating emotional valence within whinnies. To our knowledge, this is the first demonstration of perception of changes linked to emotional valence within a given vocalisation type in a non-human species, and is similar to perception of affective prosody in humans (i.e. paralinguistic emotional information in speech, which differs from discrimination of laughter and crying). This ability might enable fine-tuned communication between horses within a given situation.

### Valence perception

We previously found that whinnies produced in negative situations (i.e. separation from group member(s)) were longer (mean Dur = 2.23 s) and had a higher mean fundamental frequency (G0Mean = 1588.52 Hz) compared to those produced in positive situations (i.e. reunion with group member(s); mean Dur = 2.14 s, mean G0Mean = 1392.41 Hz [24]). Our playback experiment now confirmed that this variation in duration and frequency within whinnies produced in different contexts can be perceived, at least in the whinnies of familiar horses. Previous studies of responses to different call types produced in negative and positive contexts showed that these call types can be discriminated and that the emotion they convey can be transmitted (e.g. rodents [34], marmosets [7]). Our results indicate that perception of emotional valence is also possible within a given vocalisations type, in the same way as what has been shown for emotional arousal (e.g. [32, 45]). Separation and reunion whinnies could constitute acoustically graded variants with distinct functions, thus increasing horse apparent vocal repertoire size and potential to transmit information. More generally, we suggest that within-call type variation could enable fine-tuned communication between individuals within a given situation, unlike between-call type variation, which is related to different contexts.

In addition to the difference in reaction to familiar separation and familiar reunion whinnies that we observed, we found that the acoustic parameters of whinnies affected horses’ response in a continuous way, independently of the familiarity of the caller. Indeed, the same physiological and behavioural parameters (i.e. those loading on PC2) that differed in reaction to separation and reunion whinnies, were also affected by the parameters of the calls themselves (F0, G0 and Q25%). This suggests that the response of receivers to the negative and positive graded whinny variants is also graded. Similar results have been found by zebra finches; in addition to differences between the physiological and behavioural responses of females to the calls of their mates produced during corticosterone treatment and to regular contact calls, some of the acoustic parameters of the calls affected females’ corticosterone concentrations in a continuous way [32]. Whether horses categorise separation and reunion whinnies as the same or different call types, and what is the minimum acoustic variation that they can perceive, could be tested using further playback experiments (e.g. habituation-dishabituation paradigm [46, 47]).

Interestingly, horses’ behaviour and physiology significantly differed between playbacks of separation and reunion whinnies when these sounds were produced by familiar individuals (i.e. housed in the same farm), but not when produced by unfamiliar ones (i.e. housed in different farms), suggesting better perception of emotional valence in familiar compared to unfamiliar whinnies. Several phenomena could explain these results. First, horses could have perceived the emotional content of unfamiliar whinnies, but without reacting differently to separation and reunion whinnies, because the perceived difference might not have been meaningful to them [48]. Second, unfamiliar whinnies could be generally perceived as more negative than familiar whinnies, independently of the valence that they convey, because of the potential aggressive interactions that accompanies encounters between two unfamiliar horses [49]. This hypothesis is supported by the fact that horses’ reaction to unfamiliar whinnies suggests a negative state of high arousal; compared to playbacks of familiar whinnies, when hearing unfamiliar whinnies, horses moved more and had shorter inter-pulse-intervals (RR, i.e. higher heart rate), which indicates high arousal, and they had their head high for a longer duration, which indicates a negative emotion [24]. Third, emotional perception could be easier between individuals that are familiar with each other, notably as a result of past experiences [13]. In humans, although emotion recognition is cross-cultural, it is more accurate within cultures, due to cultural variations acquired through social learning [50, 51]. We could thus hypothesise that variation in the acoustic structure of whinnies between negative and positive situations can only be perceived by horses if they are familiar with the voice of the producer and have learned the range of changes that can occur in the producer’s vocalisations.

An additional potential explanation for our results is that emotional perception could be stronger between familiar horses, because social affiliates are generally more empathic towards each other [13]. Enhanced emotion perception or emotional contagion between social affiliates seems widespread in the animal kingdom [10, 13]. For example, corticosterone resonance occurs between female zebra finches and their pair mate, while calls from unfamiliar males do not have such clear effect [32]. Micheletta et al. [52] found that crested macaques (*Macaca nigra*) attend more to playbacks of recruitment alarm calls if these are produced by close social affiliates. Rukstalis and French [53] revealed a decrease in stress (urinary cortisol levels) linked to isolation in marmosets when playing back contact calls of their pair mate, but not when playing back calls of an unfamiliar opposite sex individual. Similar enhanced reactions to the emotions experienced by familiar compared to unfamiliar individuals have also been highlighted in studies focussing on other sensory modalities than audition (e.g. [8, 54]; review [13]). At an ultimate level, enhanced emotion perception between social affiliates facilitates reciprocal altruism, which predicts a return of favour [55].

### Emotional contagion

Emotional contagion occurs when the producer’s emotion is transmitted to the receiver. The whinnies used in our study were recorded as part of a previous study aimed at finding indicators of emotions [24]. This study demonstrated that respiration rate and time spent moving were the best indicators of emotional arousal (indicated by heart rate), while time spent chewing (i.e. moving the lower jaw in a chewing motion, without food) and time spent with the head high were the best indicators of emotional valence. If emotional valence matching had occurred during our playback experiment, we would have expected horses to have the head high for a longer duration (indicator of negative emotion) during playbacks of separation whinnies and to display more chewing motion (indicator of positive emotion) during playbacks of reunion whinnies. If emotional contagion was driven by the acoustic parameters of the whinnies, we would also have expected the time spent with the head high to increase, and the time spent chewing to decrease, with an increase in the duration and G0 of the calls played back, as predicted during a change from positive to negative emotions. Instead, chewing loaded highly on the third principal component of the PCA, which was neither affected by the valence nor by the familiarity of the calls, and the time spent with the head high was correlated positively with the scores of the first and second principal components (PC1 and PC2), which were higher during familiar reunion (i.e. positive) compared to familiar separation whinnies (i.e. negative). In addition, PC2 (indicating a higher proportion of time spent with the head high) was negatively affected by the third component of a PCA carried out on the parameters of the vocalisations. This effect indicated that whinnies that had lower fundamental frequencies (both F0 and G0), were less modulated in amplitude (AMVar) and had a higher first quartile of energy (Q25%) triggered a higher proportion of time spent with the head high. Although a low AMVar and a high Q25% suggested a negative emotion in our previous study (i.e. were significantly lower and higher, respectively, in the negative compared to the positive context), a low G0 and F0 indicated a positive emotion (particularly G0 [24]). Therefore, there is no clear evidence suggesting that contagion of emotional valence occurred during our playbacks.

One explanation for the higher proportion of time spent with the head high during familiar reunion compared to familiar separation whinnies is that horses could have been frustrated to hear reunion whinnies without seeing their group mate(s) arriving. However, frustration is a negative emotion that is likely to be of high arousal (e.g. [23]), and the responses of the horses to familiar reunion whinnies was also characterised by low respiration rates (negatively correlated with PC2), indicating low arousal [24]. Alternatively, the time spent with the head high could, in addition to indicate negative emotions in a normal situation, indicate a high level of attention to the playbacks in our experiment. This suggests that this behavioural parameter might not constitute a reliable indicator of valence in a playback situation. Further studies investigating emotional contagion through vocalisations could include preference tests (e.g. [56]), in order to know if horses judge separation whinnies as negative, and reunion whinnies as positive.

One reason for the lack of evidence for emotional contagion in our study could be that the emotion elicited in the producers by the situations during our recordings (i.e. when valence indicators were established) was stronger than the emotion triggered in the receivers during the playbacks. This could be due to the fact that the receivers of the playbacks were in a different situation than the producers of the whinnies, resulting in incongruent or weaker emotional reactions. Alternatively, emotional contagion could have occurred, but be expressed through other parameters than the ones measured in our study (e.g. facial expressions [57], odours [3]).

### Familiarity

Our results revealed increased emotional arousal (shorter inter-pulse intervals, i.e. higher heart rate; and more movements [24]) when hearing unfamiliar compared to familiar whinnies. Horses also had their head high for a longer period of time during playbacks of unfamiliar compared to familiar whinnies, which could indicate negative emotion (but see above). Encounters between unfamiliar horses can elicit aggressive interactions while the hierarchy is being established [49, 58]. The higher arousal elicited by unfamiliar compared to familiar whinnies might thus result from anticipation of such potential aggressive encounter. Similar differences in response to playbacks of unfamiliar and familiar whinnies have been observed previously in horses [38]. Lemasson et al. [38] showed that the angle of head rotation and level of postural alertness increased when the familiarity with the horse that produced the whinny decreased (group member < familiar non-group member < unfamiliar). The evidence thus suggests that horses can perceive the difference between familiar and unfamiliar whinnies, and even categorise conspecifics into several degrees of familiarity [38]. Individual vocal signatures present in whinnies might allow them to perform this sound categorisation [38]. This ability is widespread in the animal kingdom [30, 59], and can result from habituation. Within natural settings, it could enable horses to identify group members, with whom they establish long-term bonds, unlike members of other groups that are only met temporarily and that might represent a threat (e.g. competitor) [60].

## Conclusions

Although we did not find clear evidence for contagion of emotional valence, our results show that horses have the ability to perceive information about emotional valence within familiar whinnies, similarly to perception of affective prosody in humans. In addition, we show that the acoustic parameters of separation and reunion whinnies affect the physiology and behaviour of conspecifics in a continuous way. These two graded whinny variants could constitute functionally distinct calls, increasing the horses’ potential to transmit information and enable fine-tuned communication between individuals within a given situation.

## Methods

### Subjects and management conditions

Eighteen horses of various breeds, sex and age were tested in July and August 2013 (Additional file 1). All horses had been in their respective farms for at least 6 months (3–4 horses per farm). At night the horses were housed in single boxes (*N* = 4) or in boxes with paddocks, either individually (*N* = 9), or in groups of two to three horses (*N* = 5). During daytime, they were kept outdoors, either individually in adjacent fields allowing physical, visual and acoustic contact (*N* = 5), or in groups of two to four horses (*N* = 13). Horses from different farms had never encountered each other.

### Playback treatments

The separation and reunion whinnies used to build the playback treatments had been recorded in May and June 2013 from the same horses, as part of an experiment on physiological, behavioural and vocal indicators of emotions [24]. The acoustic structure of these whinnies differed significantly. For details about these situations, how underlying emotions were validated using physiological and behavioural measures, and acoustic differences between separation and reunion whinnies, see [24].

Each playback comprised a sequence of three whinnies from the same horse, with 13.5 s of silence between each whinny (15 s on average for one call and the subsequent silence interval), in order to allow horses time to react to each whinny. Preparation of sequences involved selecting the three best quality whinnies (low level of background noise) from 13 horses that had vocalised the most in our previous study [24], scaled to a relative absolute peak amplitude of 0.99, and pasted successively using Praat 5.3.41 [61]. The number of horses used to prepare playbacks was maximised so that each horse was played to no more than four subjects, either as familiar or unfamiliar treatment (each horse was played to 2.92 ± 0.86 subjects; range = 2–4). Additionally, within a farm, the same familiar horse was played to no more than two subjects, and unfamiliar horses differed for each subject. In the few cases (*N*  = 5/26 sequences) where it was not possible to obtain 3 different good quality whinnies to prepare a sequence, the same whinny was repeated three times. All sequences were then rescaled to the same maximum amplitude.

### Playback procedure

The four treatments were played once to each subject individually over two consecutive days, with two treatments per day between 9 am and 5 pm. They were broadcast in an order that was counterbalanced within horses for valence (negative = “-”, positive = “+”) and between horses for familiarity (familiar = “F”, unfamiliar = “U”; e.g. Horse 1, Day 1: F+, U-/Day 2: F-, U+; Horse 2, Day 1: U- F+/Day 2: U+ F-). Within a farm, for each of the 2 days, horses were tested one by one in the same order, with the first treatment followed by the second after about 1 h (interval between two treatments: 58 ± 12 min, range = 35 min to 1 h 30 min). To minimise behavioural reactions that would not be due to the playbacks (e.g. reaction to social separation), the subjects were tested in their home pen. The other horses from the farm (2–3 other horses for each farm) remained in their home pen also, but their view was totally occluded from the subject behind doors and fences before the playback started. This procedure did not seem to affect the normal behaviour of the horses.

The subject was equipped with the heart-rate monitor (see Physiological measures) and left for 5 min, undisturbed for habituation, which allowed the animals to return to normal activities. At the end of 5 min, the playback started. Sounds were broadcast with an Edifier S2000v loudspeaker (frequency response: 20Hz - 20 kHz), connected to a laptop where the sounds were stored in WAV format, at a sampling rate of 44.1 kHz and a bit rate of 705 kbps. Before the test started, the loudspeaker was placed behind a fence or door, at 5 m on average from the subject. To reduce habituation, the loudspeaker’s location was randomly changed between conditions for each horse. Sounds were played at an intensity estimated to be normal for horses (85.19 ± 2.38 dB measured at 1 m using a sound level meter, C weighting (SoundTest-Master, Laserliner, UK)) [38, 42]. Playbacks stopped 30 s after the end of the last whinny, and the heart-rate monitor was removed from the subjects.

### Physiological measures

Physiological measures were collected using a wireless non-invasive monitor (MLE120X BioHarness Telemetry System, Zephyr) [62, 63], fixed to a surcingle placed around the subject’s heart girth. ECG gel was applied to the electrodes before each use. The data (continuous ECG trace, breathing wave, i.e. inhalation/exhalation cycle, and skin temperature) were transmitted and stored in real time to a laptop using LabChart software v.7.2 (ADInstrument) for later analyses. During tests, one experimenter entered comments in the software indicating when each of the three whinnies of the playback sequence was broadcast. This allowed us to measure the following physiological parameters precisely for up to 10 s (when possible, i.e. good quality signal, clearly visible heart beats on the ECG trace and respiration on the breathing wave) following the beginning of each call played back (selection duration = 8.05 ± 1.87 s): inter-heart-beat interval (RR), respiration rate and skin temperature (Table 1). Such short selections allowed us to identify short-term changes in physiology in reaction to the calls played back [64, 65]. For each selection, we ensured the software tracked the heart beats (ECG trace) correctly (as displayed by event markers on the screen) and the inspiration–exhalation cycles (breathing wave). Parts of the ECG trace when atrio-ventricular blocks could be observed (i.e. one heart beat missing every 3–4 beats) where excluded [66]. Then, the inter-heart-beat interval (RR), respiration rate (breaths/s, RespRate) and skin temperature (°C, SkinT) averages were then obtained automatically from the software. These three physiological parameters had been previously shown to be affected by emotional valence and/or arousal [24]. For 15 calls played back, the quality of the signal was not good enough to extract the physiological parameters. In addition, one group mate whinnied during one call of one playback, and we omitted the physiological response of the subject to this call. In total, we were thus able to obtain the physiological parameter values in response to 200 calls played back from a total of 216 (i.e. 18 subjects*4 playbacks*3 calls).

### Behavioural measures

All tests were filmed using a Canon Legria FS2000 camcorder by an experimenter situated away from the loudspeaker. The behavioural parameters (Table 1) were scored from the videos of the tests using Interact software v. 9.0.7 (Mangold International GmbH, Arnstorf, Germany) for 15 s following the beginning of each played back call. They were scored either as occurrence (for discrete behaviours, indicated by "(min-1)" in Table 1) or as duration (for continuous behaviours). We then divided these values by the total scoring time for each call (15 s), hence obtaining frequency of occurrence for discrete behaviours (i.e. number of events per minute), and the proportion of time spent performing the behaviour for continuous behaviours. Analyses were carried out on these frequencies of occurrence or proportions. We considered for the analyses the five behaviours which were previously shown to be affected by emotional valence and/or arousal, and which had been observed during the playbacks (i.e. all except “Returns”; way and back movements along the fence or turns inside the stable [24]). In addition, we included in our analyses the latency to respond to the playbacks (see list, abbreviations and definitions of the parameters in Table 1). Because one group mate whinnied during one call of one playback, we omitted the behavioural response of the subject to this call and thus obtained behavioural data in response to 215 calls from a total of 216 (i.e. 18 subjects*4 playbacks*3 calls).

### Vocal parameters

In order to test the effect of the vocal parameters of the calls played back on horses’ physiological and behavioural responses, we analysed all the whinnies used in the experiment (*N* = 68 different whinnies) following [24]. In the same way as for the physiological and behavioural parameters, we analysed the 12 vocal parameters that were significantly affected by emotional valence and/or arousal in our previous study ([24] see list, abbreviations and definitions in Table 1). These parameters were extracted using a custom built program in Praat, which batch processed the analyses and the exporting of output data [67]. In order to prevent biases linked to the settings used for the analyses, the same settings were used to analyse both negative and positive whinnies of each producer (for details about the setting used, see [24]). G0 could not be measured in three whinnies of one producer. All the other parameters could be extracted from the 68 whinnies.

### Statistical analysis

We first tested the effect of the valence and familiarity of the calls played back on the physiological and behavioural responses of the horses (raw data are available in Additional file 5). We used a principal component analysis (PCA; prcomp function, library stats in R software 3.3.1.) to eliminate redundancy due to intercorrelation of the physiological and behavioural parameters [48]. To control for confounding factors that could have impacted on horses’ responses, instead of including the original parameter values in the PCA, we included the residuals extracted from linear models (LMs, lm function in R) fitting the following control factor: 1) age of subjects (7–23 years old, Additional file 1); 2) because each subject was tested four times and could potentially hear and habituate to the calls played to the other horses, we also included the order of the playbacks for each farm (1–12 or 1–16 playbacks depending on the farm). The resulting residuals were independent of these factors and better approximated a normal distribution, after using a log transformation for LatenceResp and RespRate, and a logit transformation for Locomotion, HeadHigh, Chewing and VocRate (see Table 1 for abbreviations and description of the parameters). Because PCA does not handle missing data, responses to playback calls where the physiological response of the subjects (*N* = 16/216) or the behavioural response of the subject (*N* = 1/216) were missing (see above for reasons) were excluded (total included in the PCA = 200 data points from 18 horses).

The principal components with an eigenvalue greater than 1 (Kaiser’s criterion) were extracted from the PCA (PC1 to PC3 of a total of 9). The effects of the valence and familiarity of the calls on PC1 to PC3 scores were then tested using LMMs (lmer function, lme4 library in R). These models (one for each PC as an outcome variable) included the valence of the calls (negative or positive), the familiarity of the calls (familiar or unfamiliar) and the interaction effect between familiarity and valence, as fixed factors. In addition, because the sex of the producer of the calls in respect to the sex of the subject might affect the responses, we added a fixed factor indicating whether the calls played back were produced by an individual of the same sex as the subject or not, as well as interaction terms between this factor and familiarity and valence. The inclusion of non-significant interaction terms in models makes the interpretation of main effects problematic [68]. On the other hand, model simplification, in which non-significant terms including interactions are dropped from the full model can lead to type 1 errors [69]. In order to be able to interpret main effects while leaving non-significant interactions in our models, we changed the contrasts of our factors (valence, familiarity and sex) from treatment contrasts (used by default by R) to sum contrasts [70]. In order to account for dependencies between data, our models included the following random effect; the playback number (each playback consisted of three calls), nested within the day of the playback (two playbacks per day), nested within the subject identity, nested within the farm where they were housed, crossed with the identity of horses whose whinnies were being played.

We then tested the effect of the vocal parameters of the calls played back on the physiological and behavioural responses of the horses (raw data available in Additional file 5). We first used a PCA in order to eliminate redundancy due to intercorrelation of the vocal parameters. To better approximate a normal distribution, we log-transformed beforehand all the vocal parameters except F0Mean and AMVar (see list in Table 1). Because the aim was to test the effect of the extracted PCs (hereafter “PCv”) on the PCs corresponding to the physiological and behavioural responses of the horses, we excluded the acoustic data for which no response was available (*N* = 16/216 data points). In addition, because PCA does not handle missing data, two additional whinnies in which G0-parameters could not be measured were excluded (Total included in the PCA = 198 data points from 18 horses). The principal components with an eigenvalue greater than 1 (Kaiser’s criterion) were extracted from the PCA (PC1 to PC4 of a total of 12; hereafter “PC1v to PC4v”). The effects of PC1v to PC4v on PC1 to PC3 scores corresponding to the physiological and behavioural responses of the horses to the playbacks were then tested using LMMs (lmer function, lme4 library in R). These models (one for each PC as an outcome variable) included PC1v, PC2v, PC3v and PC4v as fixed factors, and the same random factors as listed above (playback number within day within subject within farm crossed with producer identity).

We checked the residuals of the models graphically for normal distribution and homoscedasticity [71]. *P*-values (PBmodcomp function, pbkrtest library in R), model estimates and confidence intervals (bootMer function, lme4 library in R), were calculated using parametric bootstrap methods (1000 bootstrap samples). To this aim, models were fitted with maximum likelihood. *P*-values calculated with parametric bootstrap tests give the fraction of simulated likelihood ratio test statistic values (LRT) that are larger or equal to the observed LRT value. This test is more adequate than the raw LRT test because it does not rely on large-sample asymptotic analysis and correctly takes the random-effects structure into account [72]. When an interaction effect was significant, we carried out Tukey post-hoc tests (glht function, multcomp library in R). The significance level was set at α = 0.05. In addition, we calculated marginal (*R* ^*2*^_GLMM(m)_) and conditional *R* ^2^ (*R* ^*2*^_GLMM(c)_) of our models following [73]. *R* ^*2*^_GLMM(m)_ corresponds to the proportion of variance explained by the fixed factors alone, while *R* ^*2*^_GLMM(c)_ corresponds to the proportion of variance explained by both the fixed and random factors [73]. These two values were calculated for the full models, as well as for significant factors by including the significant factors and random effects only.

## Additional files

Additional file 1: Characteristics of the horses used in the experiment. (DOCX 13 kb)
Additional file 2: Results of the models testing the effects of the valence and familiarity of the calls broadcast, as well as of the sex of the horses, on their responses to the playbacks. (DOCX 14 kb)
Additional file 3: Model estimations corresponding to the effects of valence, familiarity and sex on the responses of the horses to the playbacks. (DOCX 14 kb)
Additional file 4: Results of the models testing the effect of the vocal parameters of the calls broadcast on the horses’ responses. (DOCX 14 kb)
Additional file 5: Raw data (physiological and behavioural responses of the horses to the playbacks, along with parameters of the vocalisations broadcast). (XLSX 52 kb)

## Abbreviations

AMExtent (dB): Mean peak-to-peak variation of each amplitude modulation
AMVar (dB/s): Cumulative variation in amplitude divided by the total whinny duration
Chewing: Proportion of time spent chewing (i.e. moving the lower jaw up and down in a chewing motion). This behaviour is performed without the presence of food in the mouth
Dur (s): Duration of the whinny
F0Max (Hz): Maximum F0 frequency value across the whinny
F0Mean (Hz): Mean F0 frequency value across the whinny
F0Start (Hz): Frequency value of F0 at the start of the whinny
G0Max (Hz): Maximum G0 frequency value across the whinny
G0Mean (Hz): Mean G0 frequency value across the whinny
G0Start (Hz): Frequency value of G0 at the start of the whinny
HeadHigh: Proportion of time spent with the eye line above the tip of the shoulder
HeadMov (min-1): Number of rapid head movements per minute
LatenceRes: Latency from the onset of the call played back to the first behavioural response (including all the above described behaviours)
Locomotion: Proportion of time spent moving (walk, trot or canter)
Q25% (Hz): Frequency value at the upper limit of the first quartiles of energy
Q50% (Hz): Frequency value at the upper limit of the second quartiles of energy
Q75% (Hz): Frequency value at the upper limit of the third quartiles of energy
RespRate (breaths/s): Respiration rate
RR (ms): Inter-heart-beat interval
SkinT (°C): Skin temperature
VocRate (min-1): Number of vocalisations (whinnies or nickers) per minute
