# Supplementary material for: Perception of emotional valence in horse whinnies
Source: Front Zool. 2017 Feb 11;14:8. doi: 10.1186/s12983-017-0193-1 (PMC5303229; doi:10.1186/s12983-017-0193-1)
Supplement: Additional file 1: — Characteristics of the horses used in the experiment. (DOCX 13 kb) [file 12983_2017_193_MOESM1_ESM.docx]

**Additional file 1.** Characteristics of the horses used in the experiment; breed, sex (F = female; G = gelding) and age (years old in 2013).

| **Farm** | **Horse** | **Breed** | **Sex** | **Age (years)** |
| --- | --- | --- | --- | --- |
| 1 | 1 | Swiss Pony | G | 20 |
|  | 2 | Swiss Halfbred | F | 23 |
|  | 3 | Swiss Halfbred | G | 7 |
| 2 | 1 | Swiss Halfbred | F | 16 |
|  | 2 | Swiss Halfbred | F | 15 |
|  | 3 | Swiss Halfbred | F | 7 |
|  | 4 | Irish | F | 23 |
| 3 | 1 | Akhal-Teke | F | 21 |
|  | 2 | Dartmoor Pony | F | 9 |
|  | 3 | Camargue Horse | F | 14 |
|  | 4 | Quarter Horse | F | 12 |
| 4 | 1 | French Saddlebred | G | 23 |
|  | 2 | Welsh Poney | G | 12 |
|  | 3 | Swiss Halfbred | F | 7 |
| 5 | 1 | Swiss Halfbred | F | 10 |
|  | 2 | Swiss Halfbred | G | 10 |
|  | 3 | English Thoroughbred | F | 22 |
|  | 4 | Swiss Halfbred | G | 19 |
