# Supplementary material for: Perception of emotional valence in horse whinnies
Source: Front Zool. 2017 Feb 11;14:8. doi: 10.1186/s12983-017-0193-1 (PMC5303229; doi:10.1186/s12983-017-0193-1)
Supplement: Additional file 2: — Results of the models testing the effects of the valence and familiarity of the calls broadcast, as well as of the sex of the horses, on their responses to the playbacks. (DOCX 14 kb) [file 12983_2017_193_MOESM2_ESM.docx]

**Additional file 2.** Statistical results (linear mixed-effects models, parametric bootstrap test); effect of the various factors (Valence, Familiarity and Sex) and their interactions on the three principal components (PC1-PC3) extracted from the principal component analysis carried out on behavioural and physiological responses of the horses to the playbacks (see Table 2 for factor loadings). Marginal (*R^2^*_GLMM (m)_) and conditional (*R^2^*_GLMM (c)_) *R^2^* of the models are given at the bottom of the table. Significant results appear in bold.

| **Response variable** | **Factor** | ***P-*value** |
| --- | --- | --- |
| **PC1** | Valence | 0.68 |
|  | **Familiarity** | **0.022** |
|  | Sex | 0.63 |
|  | Valence:Familiarity | 0.88 |
|  | Sex:Valence | 0.78 |
|  | Sex:Familiarity | 0.55 |
|  | ***R^2^*_GLMM(m)_ %** | **3.69** |
|  | ***R^2^*_GLMM(c)_ %** | **62.07** |
| **PC2** | Valence | 0.08 |
|  | Familiarity | 0.33 |
|  | Sex | 0.52 |
|  | **Valence:Familiarity** | **0.044** |
|  | Sex:Valence | 0.55 |
|  | Sex:Familiarity | 0.13 |
|  | ***R^2^*_GLMM(m)_ %** | **8.91** |
|  | ***R^2^*_GLMM(c)_ %** | **40.50** |
| **PC3** | Valence | 0.34 |
|  | Familiarity | 0.93 |
|  | Sex | 0.98 |
|  | Valence:Familiarity | 0.14 |
|  | Sex:Valence | 0.2 |
|  | Sex:Familiarity | 0.38 |
|  | ***R^2^*_GLMM(m)_ %** | **3.92** |
|  | ***R^2^*_GLMM(c)_ %** | **9.47** |
