# Supplementary material for: Perception of emotional valence in horse whinnies
Source: Front Zool. 2017 Feb 11;14:8. doi: 10.1186/s12983-017-0193-1 (PMC5303229; doi:10.1186/s12983-017-0193-1)
Supplement: Additional file 3: — Model estimations corresponding to the effects of valence, familiarity and sex on the responses of the horses to the playbacks. (DOCX 14 kb) [file 12983_2017_193_MOESM3_ESM.docx]

**Additional file 3.** Model estimates for the effect of the various factors (Valence, Familiarity and Sex) on the three principal components (PC1-PC3) extracted from the principal component analysis carried out on behavioural and physiological responses of the horses to the playbacks (see Table 2 for factor loadings). Mean, lower limit of the 95% confidence interval (lo.CI) and upper limit of the 95% confidence interval (up.CI) of the model estimates are shown for each factor level.

|  |  |  | **Model estimates** | | |
| --- | --- | --- | --- | --- | --- |
| **Response variable** | **Factor** | **Level** | **mean** | **lo.CI** | **up.CI** |
| **PC1** | Valence | Positive | -0.040 | -0.692 | 0.561 |
|  |  | Negative | -0.088 | -0.725 | 0.550 |
|  | Familiarity | Unfamiliar | -0.040 | -0.587 | 0.548 |
|  |  | Familiar | -0.309 | -0.914 | 0.346 |
|  | Sex | Same | -0.040 | -0.682 | 0.552 |
|  |  | Different | -0.106 | -0.813 | 0.58 |
| **PC2** | Valence | Positive | 0.064 | -0.515 | 0.629 |
|  |  | Negative | -0.074 | -0.661 | 0.506 |
|  | Familiarity | Unfamiliar | 0.064 | -0.474 | 0.634 |
|  |  | Familiar | 0.143 | -0.408 | 0.749 |
|  | Sex | Same | 0.064 | -0.488 | 0.630 |
|  |  | Different | 0.126 | -0.465 | 0.740 |
| **PC3** | Valence | Positive | 0.002 | -0.303 | 0.263 |
|  |  | Negative | -0.066 | -0.382 | 0.225 |
|  | Familiarity | Unfamiliar | -0.040 | -0.672 | 0.562 |
|  |  | Familiar | -0.309 | -0.918 | 0.348 |
|  | Sex | Same | 0.002 | -0.258 | 0.288 |
|  |  | Different | 0.003 | -0.309 | 0.353 |
