# Supplementary material for: Perception of emotional valence in horse whinnies
Source: Front Zool. 2017 Feb 11;14:8. doi: 10.1186/s12983-017-0193-1 (PMC5303229; doi:10.1186/s12983-017-0193-1)
Supplement: Additional file 4: — Results of the models testing the effect of the vocal parameters of the calls broadcast on the horses’ responses. (DOCX 14 kb) [file 12983_2017_193_MOESM4_ESM.docx]

**Additional file 4.** Statistical results (linear mixed-effects models, parametric bootstrap test); effect of the four principal components (PC1v-PC4v) extracted from the principal component analysis (PCA) carried out on the vocal parameters of the calls played back on the three principal components (PC1-PC3) extracted from the PCA carried out on behavioural and physiological responses of the horses to the playbacks (see Tables 2 and 3 for factor loadings). Marginal (*R^2^*_GLMM (m)_) and conditional (*R^2^*_GLMM (c)_) *R^2^* of the models are given at the bottom of the table. Significant results appear in bold.

| **Response variable** | **Factor** | **Slope estimate** | **SE** | ***P*-value** |
| --- | --- | --- | --- | --- |
| **PC1** | PC1v | -0.10 | 0.06 | 0.15 |
|  | PC2v | -0.04 | 0.07 | 0.65 |
|  | PC3v | -0.11 | 0.09 | 0.31 |
|  | PC4v | 0.00 | 0.08 | 0.99 |
|  | ***R^2^*_GLMM(m)_ %** | **3.05** |  |  |
|  | ***R^2^*_GLMM(c)_ %** | **63.04** |  |  |
| **PC2** | PC1v | 0.01 | 0.04 | 0.82 |
|  | PC2v | 0.07 | 0.05 | 0.18 |
|  | **PC3v** | **-0.16** | **0.06** | **0.022** |
|  | PC4v | 0.05 | 0.07 | 0.51 |
|  | ***R^2^*_GLMM(m)_ %** | **4.97** |  |  |
|  | ***R^2^*_GLMM(c)_ %** | **40.75** |  |  |
| **PC3** | PC1v | -0.03 | 0.04 | 0.55 |
|  | PC2v | -0.04 | 0.05 | 0.53 |
|  | PC3v | -0.04 | 0.06 | 0.59 |
|  | PC4v | 0.03 | 0.06 | 0.66 |
|  | ***R^2^*_GLMM(m)_ %** | **1.24** |  |  |
|  | ***R^2^*_GLMM(c)_ %** | **12.83** |  |  |
